# Honeycomb Actuators Inspired by the Unfolding of Ice Plant Seed Capsules

**DOI:** 10.1371/journal.pone.0163506

**Published:** 2016-11-02

**Authors:** Lorenzo Guiducci, Khashayar Razghandi, Luca Bertinetti, Sébastien Turcaud, Markus Rüggeberg, James C. Weaver, Peter Fratzl, Ingo Burgert, John W. C. Dunlop

**Affiliations:** 1 Department of Biomaterials, Max Planck Institute of Colloids and Interfaces, Potsdam, Germany; 2 Cluster of Excellence “Image Knowledge Gestaltung: an Interdisciplinary Laboratory”, Humboldt University of Berlin, Berlin, Germany; 3 Wyss Institute for Biologically Inspired Engineering, Harvard University, Cambridge, Massachusetts, United States of America; 4 Institute for Building Materials (IfB), ETHZ, Zurich, Switzerland; 5 Applied Wood Materials Laboratory, Empa, Dübendorf, Switzerland; Duke University, UNITED STATES

## Abstract

Plant hydro-actuated systems provide a rich source of inspiration for designing autonomously morphing devices. One such example, the pentagonal ice plant seed capsule, achieves complex mechanical actuation which is critically dependent on its hierarchical organization. The functional core of this actuation system involves the controlled expansion of a highly swellable cellulosic layer, which is surrounded by a non-swellable honeycomb framework. In this work, we extract the design principles behind the unfolding of the ice plant seed capsules, and use two different approaches to develop autonomously deforming honeycomb devices as a proof of concept. By combining swelling experiments with analytical and finite element modelling, we elucidate the role of each design parameter on the actuation of the prototypes. Through these approaches, we demonstrate potential pathways to design/develop/construct autonomously morphing systems by tailoring and amplifying the initial material’s response to external stimuli through simple geometric design of the system at two different length scales.

## Introduction

The adsorption and desorption of water can provide the necessary energy for a variety of actuated stress generation or movement mechanisms in the plant kingdom [1–3]. The reversible opening/closing of pinecone scales upon wetting/ drying, and the swimming-like movement of wild wheat awns upon natural day-night humidity cycles, are well-known examples of such passive hydro-actuated seed dispersal strategies found in nature [4, 5].

**Fig 1:**
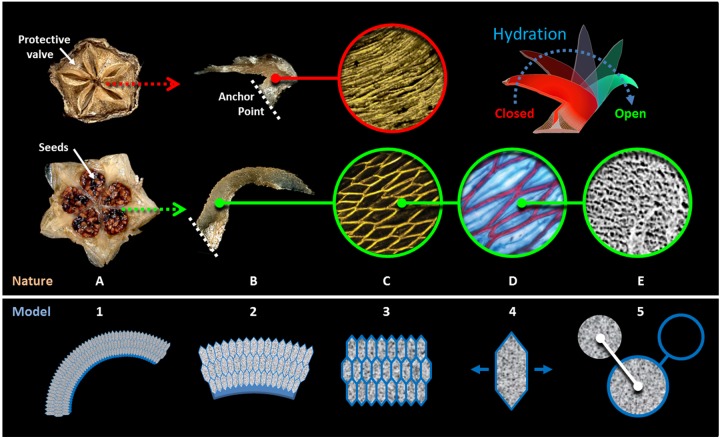
Ice plant hydro-actuation mechanism. Top: (A) The seed capsules of the ice plant, *Delosperma nakurense* are shown in dry (closed) and wet (open) state. The five seed containing compartments are closed and covered by a protective valve (each measuring ca. 3mm in length) in the dry state and open upon wetting. (B) Two hygroscopic keels halves responsible for unfolding/folding of the seed capsule; the keels are bent inward in the dry state and bend outward upon hydration. (C) Each keel consists of a network of ellipsoid/hexagonal shape cells (confocal microscopy images of the transverse cell cross-section -field diameter: ca. 0.4 mm). (D) A highly swellable cellulosic inner layer filling up the lumen of the cells is responsible for the unidirectional opening of the cells and the reversible anisotropic expansion/contraction of the cell-network upon wetting/drying cycles (FCA stained cells cross section with lignified cell wall stained in red and the cellulosic inner layer in blue- field diameter: ca. 100 μm). (E) Cryo-SEM micrograph of the cellulosic inner layer (field diameter: ca. 20μm). (redrawn after [8, 20]). Bottom: Abstraction of the actuation principles from lower to higher lengthscale; (5) volume change of a highly swellable material inside a circular confinement induces an isotropic volume change of the cell; (4) Tailoring the geometry of such cell enables an anisotropic deformation upon swelling/shrinkage cycles; (3) Through periodic arrangement of the cells, the cooperative anisotropic deformation of individual cells results in a unidirectional expansion/contraction of the cellular structure at a larger length scale,(2,1) which can be translated into bending of the whole honeycomb structure when the deformation is restricted at one side (re-sketched after [20]).

Another example of a passive actuation system is the hydro-responsive unfolding of the pentagonal ice plant seed capsules. Some species of the Aizoaceae, also known as ice plants, have evolved a sophisticated seed dispersal mechanism in which their fruit undergoes a reversible origami-like unfolding upon sufficient hydration [6, 7], the underlying biomechanics and physical chemistry of which were explained in our previous works [8, 9]. The engine of the investigated movement was found to be the water adsorption and swelling of the cellulosic inner layer (CIL) of the cell wall of the hygroscopic keel cells. The complex large-scale movement, however, could only be explained in terms of the sophisticated hierarchical design of the entire capsule (Fig 1). At a smaller length scale, and due to the confinement of the ellipsoid-hexagonal shape of the keel cells, the isotropic swelling of the CIL is translated into a unidirectional deformation along the short axis of the cells (Fig 1C). This mechanism can be defined as the "swelling honeycomb" principle. At a larger length scale, the keel is effectively a bilayer analog of a bimetallic strip [10], where the cellular structure plays the role of the “active” (expanding/contracting) element while the backing tissue is the “passive” (resistive) element, and the entire “honeycomb-backing” system bends upon differential swelling/shrinkage of the two layers (Fig 1B). Thus, such a bending mechanism at a larger scale ensures the transition from the unidirectional expansion of the cells to the unfolding of the capsule. Passive actuation systems such as these that do not depend on the active role of living cells are particularly good candidates for biomimetic transfer and further development of such autonomous ‘smart’ systems [1–3, 11–17] and as we have shown previously, the anisotropy and magnitude of the cell deformation can be tuned by appropriately choosing the shape of the confining cell, thus obtaining diverse expansion behaviors [18, 19].

In this work, we abstracted these principles to develop two types of autonomously deforming honeycombs, extending from our previous studies in this field [20, 21]. Our aim here was to quantify the actuation of these honeycombs as a result of their geometry and the mechanical and physico-chemical properties of their constituents.

In one case, we developed an analytical model that can predict the honeycomb’s deformation behavior upon swelling. This model was validated through swelling experiments on physical samples obtained through the use of high resolution multi-material 3D printing. In a second approach, we implemented the bilayer actuation principle directly into the walls of a honeycomb, creating a so-called "bilayer honeycomb" prototype and assessed their actuation behavior upon changes in environmental humidity. Further, we quantified their actuation behavior and compared these results to their corresponding analytical and numerical predictions.

## Materials and Methods

### Swelling-Honeycomb Prototype

To investigate the influence of shape and material composition on the actuation behavior of a swellable honeycomb, one needs precise control over the materials, geometry and solvent response properties. An ideal configuration would integrate highly swellable materials (like hydrogels) with the precisely defined shape of the honeycomb cells. While hydrogels could be the material of choice exhibiting high swellability, their fabrication in precisely shaped bulk objects can be challenging. One drawback of this approach is the technical difficulty of combining the hydrogel filling with the honeycomb, which exhibits very poor adhesion and tend to escape the honeycomb's cells upon swelling, producing non-uniform deformation of the cells [21]. To overcome this issue, we chose to fabricate our swellable honeycomb prototypes by means of multi-material 3D printing using polyvinilic and polyurethanic resins. With this approach, the stiff non-swelling walls are stably joined to the swelling soft inclusions thanks to the additive manufacturing process. Considering the chemical formulation of the inclusions' material, several commonly available organic solvents such as acetone and isopropanol were tested as swelling media with suitable chemical affinity. Although acetone could provide larger swelling expansions than isopropanol, we discarded its usage due to irreversible damage to the stiffer walls of the surrounding honeycomb. Therefore isopropanol was chosen as the swelling medium, producing the passive inner pressure required for the actuation, while ensuring experimental repeatability.

#### Geometrical design of 3D printed honeycombs with inclusions

**Fig 2:**
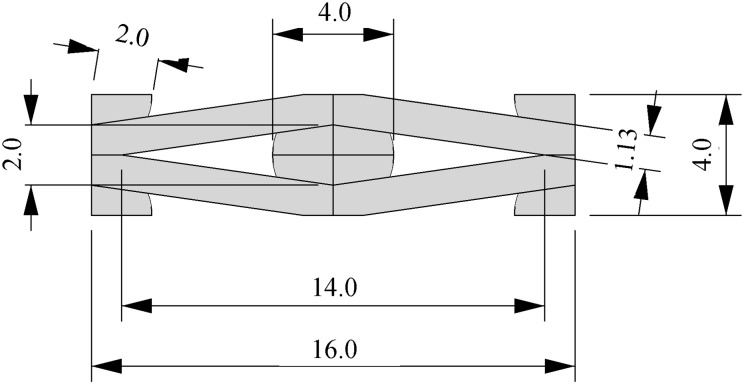
Swelling-honeycombs model. Diamond-shaped honeycombs inspired by the microstructure of the ice plant keel’s tissue. The unit cell has a diamond shape with a width-to-height ratio of 4 for stiff walls (1.13 mm thickness) and round soft inclusions. The honeycombs were built as rectangular arrays of 5-by-10 cells. All dimensions are in mm.

In our study, we used diamond-shaped honeycombs. The design was generated using the software Rhinoceros^®^ 4, (Robert McNeel & Associates) and the 2D shape of the honeycombs was obtained as an assembly of 5-by-10 cells. A single cell is shown in Fig 2 and exhibited a width-to-height ratio of 4 and a round inclusion was placed in the center of the cell. The 3D models were obtained by extruding the 2D shape by 5 mm in the z dimension. The 3D models were fabricated with a Connex500 (Stratasys) multi-material 3D printer. For the printing materials, two precursor materials RGD835 (commercially available as VeroWhite) and FLX930 (TangoPlus) were used. The soft, highly swellable FLX930 was used for the “inclusion material” (here abbreviated as MI). The stiffer, non-swallable RGD835, and three hybrid materials with intermediate properties created by combining the two precursors in different ratios (specified by Stratasys), were used for the honeycomb “wall materials” (abbreviated here as MW1, MW2, MW3, MW4; commercially available as FLX9795-DM, RGD8425-DM, RGD8430-DM and VeroWhite).

#### Fabrication of structures by 3D printing

All honeycomb samples were built with a Connex500 (Stratasys) multimaterial 3D printer from a liquid mixture of acrylate and urethane monomers and oligomers and chemical cross-linkers. The liquid mixture was deposited in a voxel-by-voxel fashion allowing fine shape control (spatial resolution of approximately 30 microns). A sweep with UV light initiates the polymerization, thus fixing the layer, which is sequentially repeated until the full 3D models have been created. Depending on the liquid precursor composition and the degree of crosslinking, a broad range of mechanical properties can be achieved from stiff duroplastic to soft rubber-like materials. The degree of crosslinking also directly influences the swelling capacity of the polymer.

#### Assessment of mechanical properties of dry 3D printed polymers

Tensile tests were performed on a Zwick/Roell^®^ testing machine, equipped with a 500 N load cell according to standard test method for tensile properties of plastics ASTM D 638. All tests were conducted at a crosshead speed of 1 mm/min at room temperature (22°C). For the tests, dog bone shaped tensile samples with neck cross sections of 3.0 by 3.4 mm and 6.0 by 3.4 mm were used. Strain was measured as the ratio between crosshead travel distance and effective grip-to-grip separation, which resulted in a 5% error in the effective strain, measured manually with a gauge in the neck region. A preload of ca. 0.5–1.0 N was applied to the samples before commencing in order to ensure that the specimen was perfectly straight prior to testing. Young’s modulus (*E*) was extrapolated as the linear regression of the stress-strain curve in a given σ_low_–σ_high_ stress interval. Assuming the material to be isotropic and incompressible in dry conditions (Poisson’s ratio ν = 0.5, a reasonable estimate for rubber-like materials [22]), the shear modulus *G* was derived via the following formula:

$$G=\frac{E}{2\left(1+\nu \right)}=\frac{1}{3}E$$

The shear modulus values of all polymers used in the experiments are listed in Table 1.

**Table 1:** Shear modulus G (in the dry state) and stretch ratio λ_0_ upon swelling in isopropanol of 3D printed polymers.

Material	MI	MW1	MW2	MW3	MW4
	FLX930 (TangoPlus)	FLX9795-DM	RGD8430-DM	RGD8425-DM	RGD835 (VeroWhite)
*G* [MPa]	0.135	0.459	2.089	96.5	176.7
*λ*_*0*_ [adim.]	1.363	1.23	1.135	1.0044	1.0033

#### Assessment of swelling of 3D printed polymers

Rapid prototyped bar-shaped samples (LxHxW: 50x5x5 mm) were soaked in isopropanol in glass petri dishes, covered with a glass lid and sealed with an O-ring. The swelling process was recorded with a camera taking snapshots every 20 minutes until a stable configuration was obtained (ca. 4 to 5 days). For the soft material samples (MI), we observed surface wrinkling in the initial stages of swelling since isopropanol diffusion was not instantaneous, and the sample surface underwent larger expansions than the core during the diffusion transient. Swelling expansion was measured at equilibrium (when the sample was uniformly swollen) in the central field of the sample with the aid of image analysis software ImageJ v1.43l [23]. Due to the large expansion caused by swelling, we adopted the stretch ratios as a measure for the swelling. This choice was maintained when developing the non-linear analytical model (see section 3.1). The free swelling stretch ratios at equilibrium—the ratio between original length and length in the fully swollen state of the polymers due to swelling–are listed in Table 1.

### Bilayer-Honeycomb Prototype

#### Material selection

For the bilayer-cell prototype, the goal was to operate within biologically relevant conditions, leading to wood as the first choice for the active element. Hence, spruce veneers with various thicknesses were utilized as the active layer since the direction of the fibers in wood provided the required swelling anisotropy. For the passive resistive element, materials with almost no swelling such as polymeric films, or materials with more isotropic response such as paper or tape were used. Based on the cooperative response of the two elements in the bending bilayer, and the simplicity of the fabrication and characterization approach, the final hydro-actuated demonstrator was made up of 0.6mm thick spruce veneers (*Picea abies* (L.) Karst.), cut through their radial planes, as the active layer, and 0.2 mm thick paper sheets (Supersilk DCP; 222200, Fischerpapier), with a negligible swelling compared to wood, were utilized as the passive/resisting layer.

#### Simple bilayer

6×2 cm pieces of spruce veneer were cut with the spruce fibers running perpendicular to the longer axis of the rectangle, and were glued (PUR Bond HBS 309 polyurethane glue) to 6×2 cm pieces of the paper cut with the longer axis being parallel to the longer side of the paper (where fibers are more aligned due to the rolling direction in paper manufacturing). Industrially made paper displays typically anisotropic mechanical and swelling properties as the rigid cellulose fibers tend to reorient along the rolling direction during the production process leading to a higher elastic modulus and a lower swellability along that production direction. The orientation of the paper (resistive layer) in the bilayer structure was thus chosen such that the paper’s rolling direction matched the longitudinal direction of the bilayer. The Spruce veneers were attached to the top of the backing paper layer with spruce fiber cells running perpendicular to the bilayer length. Hence, upon actuation cycles, the spruce veneers expanded/contracted due to the swelling/shrinkage perpendicular to its fiber direction, while the paper layer swelled less along the fiber/rolling direction and resisted the swelling of the spruce thus leading to bending of the bilayer.

#### Bilayer-cell

Two of each bilayers were made with an extra 1 cm long paper tail on both sides. Each tail was attached to the tail of the other bilayer such that the paper sides of the two bilayers faced each other with the spruce veneers making up the outer side of the final cell structure.

#### Bilayer-honeycomb

Four active elements (6×2 cm^2^ spruce veneers) were glued to 26×2 cm^2^ paper strips with 3 mm spaces between them, leaving two 1 cm tails on both ends of the paper strip free. Two of these strips were attached face-to-face end-to-end resulting in a row of four cells with the paper as their inner walls and the wood covering their outer layers. Ten of these rows of cells were then connected to each other at the middle of each cell to build up a honeycomb structure. To monitor the actuation of the prototype upon changes in relative humidity (or wetting-drying cycles), the assembled honeycomb was supported with an external frame by two wires running through the paper tails on both sides of the cell rows.

#### Analysis of the bilayer-cell hydro-actuation

To analyze the actuation of the spruce-paper bilayers and bilayer-cells, samples of each (6×2 cm^2^) were fixed horizontally in air on their tails, with the edges of the bilayer stripes facing the camera. Actuation was monitored by taking sequential images of the assembly and changes in the curvature of the bilayer stripes and the opening of the cells (distance between the outer edge of the two walls of the bilayer-cells) were measured using ImageJ v1.43l [23] in two different sets of experiments. In the first experiment, the set was moved from the initial state at room temperature (~25°C, ~50% RH) to an insulated in-house-built humidity chamber with relatively constant RH of 92–95% (~75×50×30 cm^3^, insulated box with a constant flow of humid air). In the second experiment, liquid water was sprayed on all sides of the samples, and the actuation upon wetting and the consequent drying at room condition (~25°C, ~50% RH) was monitored.

To investigate the potential of utilizing this bilayer-cell structure as a functional prototype, the bilayer-honeycomb assembly was moved from ambient conditions (~25°C, ~50% RH) into a climate-controlled room with 95%RH, where the actuation was monitored by taking pictures of the honeycomb opening/closing structure every 10 minutes for 18 hours.

#### Material characterisation

The elastic moduli and swelling coefficients of the active and passive layers were measured and used as an input for modeling the actuation of the spruce-paper bilayer and bilayer-cells. Samples of spruce veneer and paper were cut in 2×1 cm^2^ pieces in both parallel and perpendicular directions to wood fibers and the paper’s rolling direction. Unidirectional tensile tests were performed by an in-house-built bi-directional tensile testing device for three samples of each material and direction. In order to determine their elastic moduli, each sample was stretched at constant speed slightly above its yield point (100 N mini load cell, ALF259-Z3923, Kelkheim, Germany; velocity controlled mode, strain rate 10 μm s^-1^, room condition ~25°C, ~45% RH). The strain upon tension was measured by monitoring the displacement of two sets of points on the sample surface by an in-situ installed camera, and the elastic moduli were obtained by measuring the slope of the linear region of the stress-strain curve (~0.2% strain). Measurement averages for three samples of each material and each direction were calculated and further used for finite element (FE) simulation of the actuation (Table 2). The effect of the relative humidity changes (50 to 95%) on the stiffness of the two layers was assumed to be negligible, as the bilayer bending mainly depended on the ratio between the Young’s moduli of the two layers rather than their absolute values [10] and the deterioration of the stiffness due to increasing moisture content was assumed to be approximately the same for both layers at these humidity changes [24, 25].

**Table 2:** Input parameters for FE simulation of bilayer-cell actuation.

Layer	Thickness [mm]	Stiffness^a^ [MPa]	Swelling strain [%]
Active	h_1_ = 0.6	E_1_ = 284	ε_l_ = 2.23
Passive	h_2_ = 0.2	E_2_ = 5147	ε_l_ = 0.06

*assumed constant throughout simulation.

The same tensile tester was also used to measure the swelling strain of the active and passive layer (Motor position controlled, 50 mN mini load cell, 3×2 cm^2^ samples). Samples were placed between the two gages in the humidity chamber at ambient conditions (~45–50%RH), the chamber was closed and the RH was increased to 95–98% for 15 to 20 hours, and the dimensional changes of the samples upon water absorption were monitored by measuring the distance between the two set of points at two ends of the samples near the gages with a camera. The mechanical testing software was programmed to reset the force to zero upon detection of any increase in the force upon dimensional change of the samples, so that the samples were effectively swelling freely. Average swelling measurements for two samples of each material in each direction were calculated and utilized as an input for FE simulations of the actuation (Table 2).

#### Finite element simulation of the bilayer-cell actuation

The curvature (κ) of a bilayer undergoing differential swelling (Δε*) is given by Thimoshenko’s formula [10]:

$$\kappa =f\left(m,n\right)\frac{\Delta \varepsilon^{*}}{h}$$

where *h* is the total thickness of the bilayer and *f*(*m*, *n*) a function that depends on the thickness $\left(m=\frac{h_{1}}{h_{2}}\right)$ and stiffness $\left(n=\frac{E_{1}}{E_{2}}\right)$ contrast between the “active” (wood) and “passive” (paper) layers. According to the experimental prototype described in the preceding sections, the following parameters were chosen:

Using the same geometrical and material parameters, the actuation of the bilayer was simulated by the finite-element method using the commercial software Abaqus^®^. The geometry of the bilayer was discretized with eight-node linear brick elements (C3D8) staggered along the thickness of the bilayer in order to adequately capture the bending effect (ca. 10 elements). In first approximation, both layers were assumed to follow a linear elastic behavior, and the swelling was introduced into each layer as an eigenstrain (ε*, Table 2). In contrast to the swelling-honeycomb case, we adopted a small strain, large deformation assumption (nonlinear geometry), which well suits the experimental observations of small swelling strains leading to large deflections of the bilayer. Due to the elongated geometry of the bilayers and the strong difference between the swelling behaviors in the longitudinal and transverse directions, only the longitudinal behavior was considered. In the case of bilayer cells, the geometry of the connection between the two bilayers was drawn in detail. The resulting curvatures were analyzed using Rhinoceros^®^.

## Results and Discussion

### Hydro-responsive swelling-honeycomb actuators

Our first fabrication strategy directly implemented the three design principles abstracted from the study of the ice plant seed capsules. The isotropic swelling of a highly swellable material was used as the source of actuation, which was then transformed into an anisotropic actuation behavior through a diamond shaped honeycomb confinement. As a result, a unidirectional expansion was generated, which was relatively large with respect to the free swelling isotropic value.

**Fig 3:**
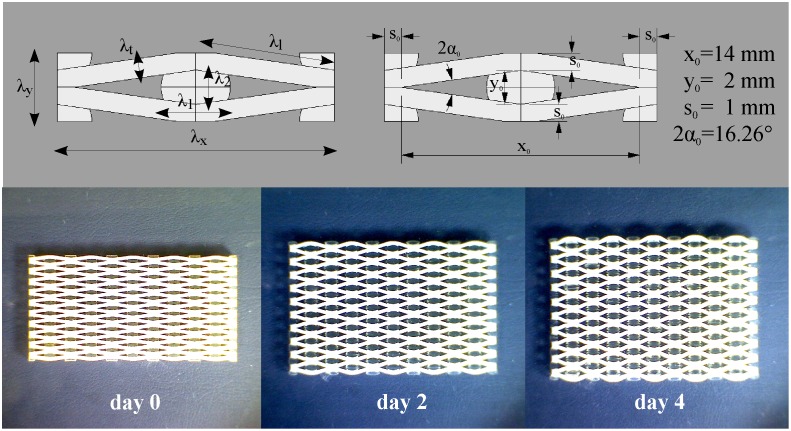
Swelling of a honeycomb with stiff (MW4) walls and soft (MI) inclusions. The honeycomb’s unit cell geometry is reported with the kinematic variables (upper left) used in the analytical model and the actual dimensions (upper right). Swelling of a diamond-shaped honeycomb with”stiff” walls (G_walls_ = G_MW4_ = 176.7 MPa) and soft cell inclusions (G_inclusions_ = G_MI_ = 0.135 MPa). Snapshots cover 4 days of swelling in isopropanol at room temperature (T = 22°).

To demonstrate this effect, we monitored the expansion of the 3D printed honeycomb samples (Fig 3) built with stiff (MW4) and soft (MI) materials for walls and cell inclusions respectively.

As the inclusions in the cells swelled, the honeycomb angle 2α (see Fig 3) between two walls increased, resulting in a highly anisotropic expansion: the honeycomb expands vertically with almost no horizontal deformation. The effect is more pronounced in a honeycomb with the same unit cell shape but having thinner walls (see S1 Movie), as thinner walls are less rigid. This simple experiment shows how the shape anisotropy of the constraining rigid framework of the walls directs the isotropic expansion of the inclusions into the vertical direction, while the wall thickness modulates the expansion magnitude. Moreover, the anisotropic honeycomb expansion is also dependent on the mismatch between the swelling properties of walls and inclusions: intuitively, a honeycomb made of a single material (for both walls and inclusions) would show isotropic expansion.

To understand the role of these parameters (the mechanical properties of the materials, the honeycomb geometry and the physico-chemical characteristics of the system: temperature, solvent properties, polymer-solvent affinity) we provide here a minimal description of the swelling-honeycomb actuators through an analytical model based on the well-known statistical theory of Flory and Huggins as formulated by Treloar [26, 27]. While three dimensional formulations of swelling in a continuum are available [28], we propose an analytical model in which the dependence from all the mentioned parameters remains explicit. The basic assumption of this theory is that swelling is driven by differences of chemical potential of the solvent that drive its diffusion in the polymeric phase. We can adopt the same assumption since the polymeric chains of the inclusions and walls cannot diffuse into each other.

Observation of the 3D printed model expansion reveals that all cells in the model deform similarly (the boundary cells deformation deviates only slightly from that in the center cells), so that the deformation of the whole system can be studied by looking at one repetitive unit cell. Denoting with *λ*_*i*_ the stretch ratio–that is the ratio between the deformed and undeformed lengths- along direction *i* (where subscripts *x*, *y*, *z* refer to the horizontal, vertical and out-of-plane axes of Fig 3; and *l* and *t* refer to the longitudinal and transverse directions of the wall) and taking superscripts “*W*” and “*I*” for walls and inclusions respectively, geometrical compatibility requires:

$$\alpha =\text{arctan}\left(\frac{y_{0}\lambda_{y}^{I}}{x_{0}\lambda_{x}^{I}}\right)$$

$$s=\frac{t_{0}\lambda_{t}^{W}}{\sqrt{1+\text{sin}\left(2\alpha \right)}}$$

$$\lambda_{l}^{W}=\lambda_{x}^{I}\text{cos}{\left(\alpha \right)}^{2}+\lambda_{y}^{I}\text{sin}{\left(\alpha \right)}^{2}$$ (1)

Then the stretch ratios *λ*_*x*_, *λ*_*y*_, *λ*_*z*_ of the composite unit cell read as:

$$\lambda_{x}=\frac{x_{0}}{x_{0}+2\;s}\lambda_{x}^{I}+\;\frac{2\;s}{x_{0}+2\;s}\;\left(\text{sin}{\left(\alpha \right)}^{2}\lambda_{t}^{W}+\text{cos}{\left(\alpha \right)}^{2}\lambda_{l}^{W}\right)$$

$$\lambda_{y}=\frac{y_{0}}{y_{0}+2\;s}\lambda_{y}^{I}+\;\frac{2\;s}{y_{0}+2\;s}\;\left(\text{cos}{\left(\alpha \right)}^{2}\lambda_{t}^{W}+\text{sin}{\left(\alpha \right)}^{2}\lambda_{l}^{W}\right)$$

$$\lambda_{z}=\lambda_{z}^{I}=\lambda_{z}^{W}$$ (2)

With this convention, only 4 variables are needed to describe the deformation state of the unit cell: the principal stretch ratios of the inclusion and the wall transverse stretch ratio (that is: $\lambda_{x,}^{I},\;\lambda_{y}^{I},\;\lambda_{z}^{I},\;\;\lambda_{t}^{W}$). To predict the swollen configuration we need 4 independent equations. With this aim, we set up the chemo-mechanical energy balance during swelling.

The chemo-mechanical balance in one material system requires that the sum of all chemical and mechanical works due to swelling of *n* moles of solvent be zero; these are: the gain in free energy $\frac{\partial \Delta G^{I}}{\partial n^{I}}$, the elastic work of the polymeric network $\frac{\partial W_{e}^{I}}{\partial n^{I}}$ and the work of external forces, which in our case is the mechanical work spent to bend and stretch the honeycomb walls $\frac{\partial W_{ext}^{W}}{\partial n^{I}}$. These terms are dimensionally energy densities and therefore have to be integrated over the corresponding volumes (that is, the walls and inclusion volumes). The resulting balance looks like:

$$V_{0}^{I}\left(\frac{\partial \Delta G^{I}}{\partial n^{I}}+\frac{\partial W_{e}^{I}}{\partial n^{I}}\right)+V_{0}^{W}\frac{\partial W_{ext}^{W}}{\partial n^{I}}=0$$ (3)

The free energy change due to swelling in a non crosslinked polymer is (note that, in our formalism $\frac{\partial \Delta G^{I}}{\partial n^{I}}$ is an energy per unit volume provided that moles *n* are defined as an non-dimensional quantity through Eq 6):

$$\frac{\partial \Delta G^{I}}{\partial n^{I}}=\frac{RT}{\upsilon_{iprOH}}\left(ln\left(1-u\right)+u+\chi u^{2}\right)$$ (4)

where *υ*_*iprOH*_ is the molar volume of isopropanol and *u* is the polymer volume fraction $u={\left(\lambda_{1}^{I}\lambda_{2}^{I}\lambda_{3}^{I}\right)}^{-1}$. The remaining mechanical quantities (elastic work spent on the inclusions polymer network $W_{e}^{I}$ and the work of external forces $W_{ext}^{I}$) depend directly on the stretch ratios $\lambda_{i}^{I}$, $\lambda_{i}^{W}$ moreover, since the solvent is incompressible, their variation due to sorption of *n* moles of solvent is more conveniently written as:

$$\frac{\partial W_{e}^{I}}{\partial n^{I}}=\frac{\partial W_{e}^{I}}{\partial \lambda_{i}^{I}}/\frac{\partial \lambda_{i}^{I}}{\partial n^{I}}$$

$$\frac{\partial W_{ext}^{I}}{\partial n^{I}}=\frac{\partial W_{ext}^{I}}{\partial \lambda_{i}^{I}}/\frac{\partial \lambda_{i}^{I}}{\partial n^{I}}$$ (5)

Where *n*^*I*^ is the volume concentration of solvent molecules in the cell material:

$$n^{I}=\lambda_{x}^{I}\lambda_{y}^{I}\lambda_{z}^{I}-1$$ (6)

and $W_{e}^{I}$ is:

$$W_{e}^{I}=\frac{1}{2}\;G^{I}\left(\lambda_{x}^{I}{}^{2}+\lambda_{y}^{I}{}^{2}+\lambda_{z}^{I}{}^{2}-3\right)$$ (7)

**Fig 4:**
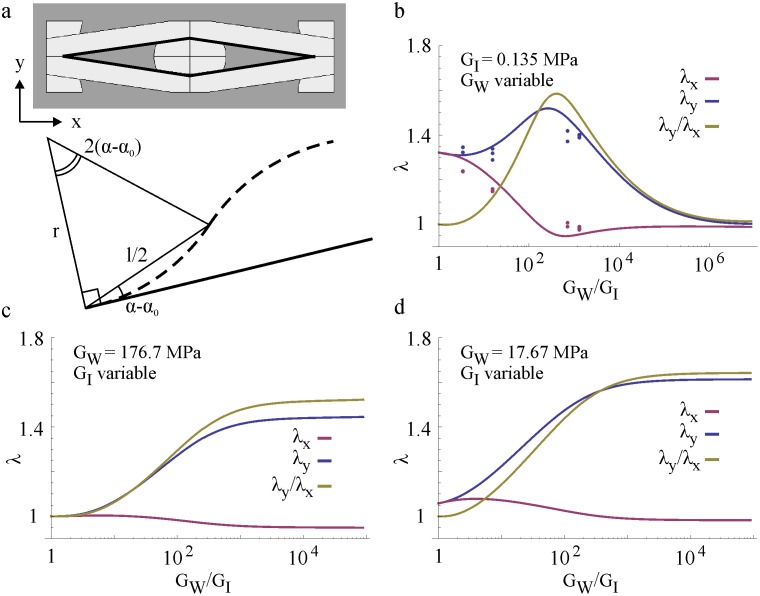
Influence of walls (G_W_) and inclusions (G_I_) rigidity on the honeycomb swelling deformations (dots: experiments; solid lines: analytical prediction). (a) Schematics to estimate the honeycomb wall curvature due to a deformation from straight (solid line) to S-shaped (dashed line). (b) Influence of increasingly rigid walls and constant stiffness inclusions (G_MI_ = 0.135 MPa) on the overall honeycomb expansion. Dots correspond to measured swelling of 3D printed honeycombs with soft inclusions (G_MI_ = 0.135 MPa) and increasingly stiff walls (respectively, G_MW1_ = 0.459, G_MW2_ = 2.089, G_MW3_ = 96.5 and G_MW4_ = 176.7 MPa). (c, d) For a fixed wall rigidity (c: G_W_ = G_MW4_ = 176.7 MPa; d: G_W_ = 0.1*G_MW4_) and increasingly softer inclusions, the (anisotropic) honeycomb expansion increases until a plateau is reached. Analytical prediction validated through experiments only for case b).

The work of external forces $W_{ext}^{W}$ is the sum of two contributions: a bending term $W_{b}^{W}$ taking into account changes in curvature of the walls (pure shape changes) and a volumetric term $W_{vol}^{W}$ that tracks pure size changes. Given that the honeycombs are translational-symmetric and they typically bend only in the cross-sectional plane, we consider the honeycomb walls as thin plates of length *l*, height *h* and thickness *t* and use the Euler-Bernoulli beam theory to estimate their bending energy:

$${\bar{W}}_{b}^{W}=\frac{1}{12}E^{W}\iint_{0,0}^{l,h}\frac{t^{3}}{r{\left(u\right)}^{2}}\;du\;dv$$ (8)

where the bar accent means that it is an integral quantity and *E*^*W*^ is the Young modulus of the walls. Here variables *u* and *v* span the plate length and height and *r* is the radius of curvature of the walls. The actual point-by-point value of *r* can be calculated by solving the so-called elastica of the beam. To achieve this, one needs to know the boundary conditions and the loads applied to the beam. To simplify the matter we assume perfectly rigid honeycomb joints (that is, the walls don’t change orientation at the ends) implying that the walls can only adopt an S-shaped bent configuration (such deformation mode is well confirmed by the experiments). If we assume a constant radius of curvature for each wall half (see Fig 4a) we can relate the radius of curvature to the deflection of the beam:

$$r=\frac{l_{0}\;\lambda_{l}^{W}}{4\;\text{sin}\left(\alpha -\alpha_{0}\right)}$$ (9)

Expliciting the dependence to the stretch ratios $\lambda_{i}^{I}$ and $\lambda_{i}^{W}$, the bending energy per unit volume of dry polymer can be expressed as:

$$W_{b}^{W}=\frac{1}{12}E^{W}\;\lambda_{0}^{W}{}^{-1}{\left(\frac{t_{0}\;\lambda_{t}^{W}}{r\left(\lambda_{x}^{I},\lambda_{y}^{I}\right)}\right)}^{2}\lambda_{t}^{W}\lambda_{l}^{W}\lambda_{z}^{W}$$ (10)

The volumetric term $W_{vol}^{W}$ tracks the elastic energy stored in the swollen walls due to mechanical work done by the inclusions and involving principal deformations. Therefore, in this term we should not consider the quota of volumetric strain induced in the walls by swelling. Practically this means that the elastic energy due to principal deformations must be diminished by an amount relative to an equivalent isotropic swollen state. This means:

$$W_{vol}^{W}=\frac{1}{2}G^{W}\;\lambda_{0}^{W}{}^{-1}\left(\lambda_{l}^{W}{}^{{}^{2}}+\lambda_{t}^{W}{}^{{}^{2}}+\lambda_{z}^{W}{}^{{}^{2}}-3\;\lambda_{0}^{W}{}^{{}^{2}}\right)$$ (11)

Where the equivalent isotropic swollen state has by definition the same polymer volume fraction:

$$\lambda_{0}^{W}={\left(\lambda_{l}^{W}\lambda_{t}^{W}\lambda_{z}^{W}\right)}^{\frac{1}{3}}.$$

Finally, substituting Eqs (4), (7), (10) and (11) through Eq (5) in Eq (3) and using *x*, *y*, *z* for *i* results in three equations embodying the equilibrium of (generalized) forces along the honeycomb principal directions.

A fourth equation derives from the chemo-mechanical equilibrium of the walls in the transverse direction *t*. In this case the term for external mechanical work can be put to zero since, by geometric construction of the cell, a transverse expansion $\lambda_{t}^{W}$ of the walls due to swelling doesn’t cause any deformation on the inclusions. Therefore this condition translates in:

$$\frac{\partial \text{Δ}G^{W}}{\partial n^{W}}+\frac{\partial W_{e}^{W}}{\partial n^{W}}=0$$ (12)

where relations similar to Eqs (4)–(7) apply along the walls’ transverse direction.

The measured honeycomb expansions and analytical predictions as function of the walls’ and inclusions’ shear modulus ratio G_W_/G_I_ are presented in Fig 4. In plot b) the solid lines are obtained by iteratively solving Eqs (3) and (12) with a fixed shear modulus (G_I_ = G_MI_ = 0.135 MPa) and a variable walls’ shear modulus increasing over several orders of magnitude, whereas the dots mark the principal extensions λ_*x*_, λ_*y*_ and the anisotropy ratio λ_*y*_/λ_*x*_ measured from swelling experiments of honeycombs with walls’ of increasingly stiffer materials (MW1, MW2, MW3, MW4, see Table 1). The theoretical predictions are in good qualitative agreement with the experimental data. Moreover, we can now clearly appreciate how the actuation of these honeycombs depends on the walls/inclusions properties mismatch. Indeed, when the walls have the same mechanical properties of the inclusions, the honeycomb deforms isotropically to the free swelling expansion of the inclusions material (MI) *λ*_*0*_ = 1.363, see also Table 1). As the walls’ rigidity increases, the effect of the honeycomb geometric anisotropy arises: a maximum extension (+50%) is achieved along the vertical direction (*y*) while a slight horizontal contraction is observed. Notice that the maximum expansion *λ*_*y*_ is larger than the free swelling expansion *λ*_*0*_: the walls effectively reduce the expansion along directions *x* and *z* to maximize *λ*_*y*_. For very rigid walls, the principal extensions tend to unity, meaning that the honeycomb doesn’t deform. From our prediction, to maximize the expansion along the honeycomb soft axis (*y-*direction) the walls material has to be 100 to 1000 times stiffer than the inclusions.

To maximize the honeycomb expansion for a given choice of wall material, one could also think of indefinitely decreasing the inclusion’s rigidity: ideally, infinitely soft inclusions would undergo the largest volume expansions, possibly producing a higher inflation of the honeycomb cells. Our model predicts that this is true until the inclusions’ shear modulus *G*_*I*_ is one thousand times smaller than *G*_*W*_ (Fig 4b and 4c); for smaller *G*_*I*_ a plateau is reached. This behavior can be understood in terms of the energy quantities at play; choosing softer inclusions, means decreasing the energy spent to elastically deform the polymeric network. Nevertheless, even if this work vanishes (using a non-cross-linked polymer for example), the amount of free energy that can be gained from swelling is limited by the physico-chemical properties of the system (represented by the molar volume of the solvent ν and the solvent-polymer affinity χ in Eq 4, see also [29]). Therefore, to additionally increase the expansion and actuation ability of such swelling-based honeycomb actuator one could choose a better solvent, more affine to the polymeric material used for the inclusions.

**Fig 5:**
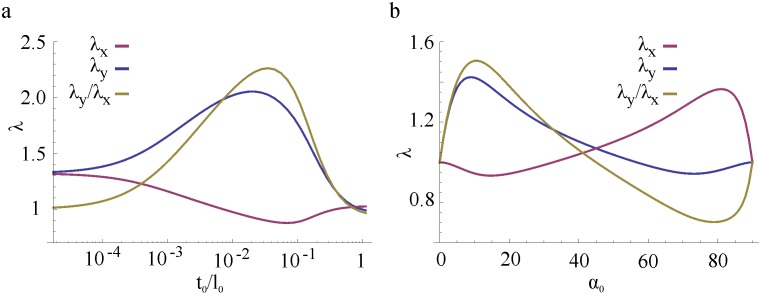
Influence of walls aspect ratio (t_0_/l_0_) and honeycomb angle (α_0_) on the honeycomb swelling deformations for G_W_ = G_MW4_ = 176.7 MPa and G_I_ = G_MI_ = 0.135. (a) Influence of walls aspect ratio on the honeycomb expansion. For walls of vanishing thickness the honeycomb expands isotropically as the inclusions. For very thick walls the honeycomb doesn‘t expand at all. (b) Influence of honeycomb angle on the honeycomb expansion.

Another way to maximize the honeycomb expansion is by controlling its shape. Through our model, we predicted the total honeycomb swelling as a function of two shape parameters, namely the walls aspect ratio t_0_/l_0_ (the ratio between thickness and length) and the honeycomb cell half-angle α_0_. The results (calculated for G_W_ = G_MW4_ = 176.7 MPa and G_I_ = G_MI_ = 0.135) are presented in Fig 5. For large thickness t_0_≈l_0_ (and therefore large flexural rigidity of the walls) the model correctly predicts zero expansion (stretch ratios *λ*_*x*_, *λ*_*y*_ tend to 1). Similarly, when t_0_/l_0_ tends to zero (that is for walls of vanishing thickness) the honeycomb expands isotropically to the free swelling expansion *λ*_*0*_ of the inclusions (in this case *λ*_*0*_ = 1.363, see also Table 1). These two extremes, explain the role of the aspect ratio of the walls: more slender walls provide less mechanical resistance and enhance the inclusions expansion, but a minimum constraining effect is needed in order to direct the inclusions expansion along direction *y*. The optimal trade-off, for our choice of other parameters, is obtained for t_0_/l_0_≈0.02 where *λ*_*y*_≈2 (100% expansion in vertical direction).

The relationship between honeycomb angle and expansion is presented in Fig 5b, where, as expected, *λ*_*x*_*(*α_0_*−45°) = λ*_*y*_*(45°-*α_0_*)* due to the geometric construction of the honeycomb cell. Low angles (α_0_<45°) maximize *λ*_*y*_: indeed at lower angles the force exerted by the inclusions on the walls has a longer lever arm (l_0_cos(α_0_)). Nevertheless, when the angle approaches zero, the volumes of the inclusions vanishes, resulting into *λ*_*x*_,*λ*_*y*_ →1. Again, there is an optimal trade-off value, which falls at α_0_≈10° for which *λ*_*y*_≈1.5.

To conclude this section, we showed how a swelling-based honeycomb actuator can be designed in terms of the most suitable choice of materials, geometry and solvent, and indicated the most important parameters that govern its expansion. Despite the fact that our model assumes a thin wall hypothesis for the bending term (this means that it neglects shear stress contributions which become dominant for thick walls), it produces reasonable estimates of the honeycomb swelling behavior. In passing, we note that the present treatment could also be used to predict the maximum force per unit area at zero-expansion that such a honeycomb actuator could exert.

### Hydro-responsive bilayer-honeycomb actuators

As an alternative configuration to the swelling-honeycomb actuation system, where the opening/closing of the cells were achieved through pressurizing the cell walls through swelling of the inclusions inside the cells, the goal here is to locate the actuation inside the honeycomb cell walls. Based on the bilayer bending movement principle seen in the flexing of the ice plant keel and other passive hydro-actuated movement in plants [4, 5, 30], the goal was to create a cell with hydro-responsive walls that change their curvature upon humidity changes, inducing the opening/closing of the cell. To obtain such a curvature change, and using the Timoshenko bi-metal strip model (Timoshenko, 1925), the cell walls can be constructed as bilayers, where the layers have different unidirectional hygroscopic coefficients [20].

At the smallest scales of the design, the goal was to translate the material’s response to an external stimulus into a unidirectional deformation of the “active element”, where the orientation of a non-deforming stiff element inside a softer extendible matrix determines the directionality of the swelling. In our case, the direction of the fibers in spruce veneers could satisfy the required anisotropy of the swelling of the active layer and introduce the unidirectional expansion in the bilayer longitudinal direction, while the underlying low swelling paper would function as the “passive layer”. A bilayer made up of two of such elements attached together would bend upon actuation to compromise between the extending active element and the resistive layer, and by attaching two of such bilayers at their ends in a mirrored fashion (passive layers facing each other), the actuated-bending of the bilayers would result in opening/closing of a “bilayer-cell” ([20], also see S2 and S3 Movies).

**Fig 6:**
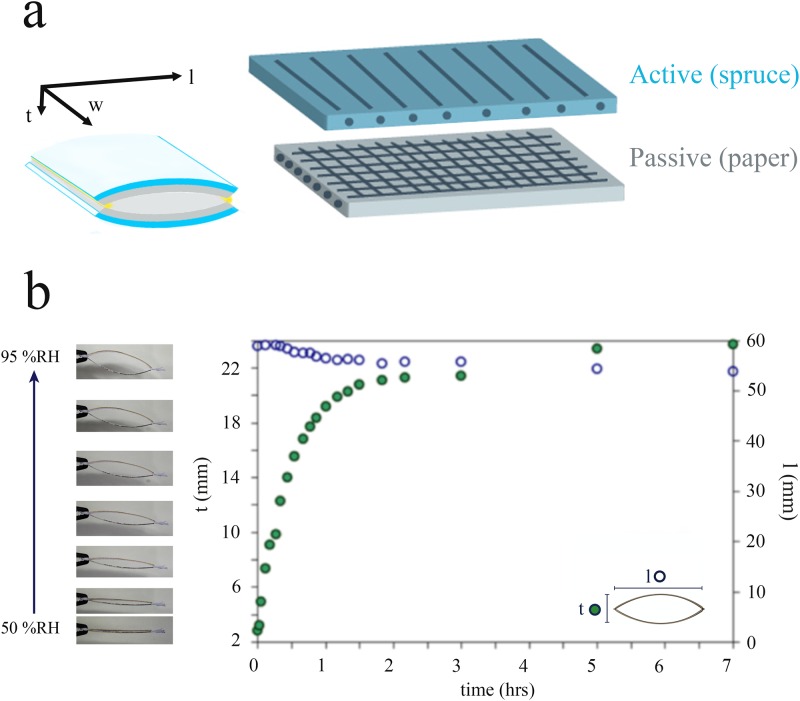
Structure and deformation of the bilayer-cell upon actuation. (a) Spatial arrangement of spruce (active) and paper (passive) layers in the bilayer-cell, with the corresponding fibers’ orientation within each layer. (b) Dimensional changes of the bilayer-cells upon exposure from initial 50% RH to a 95% RH, with the corresponding sequential images of the hydro-actuated movement (t = cell width, l = cell length).

The passive hydro-actuation and dimensional changes of the bilayer-cell were investigated by taking the cells from 50%RH at room temperature and exposing them to 95% relative humidity in an in-house-built humidity chamber (Fig 6). The bilayer-cell starts to open along their short cross-sectional axis (t) almost immediately after exposure to 95% relative humidity, and reached an 8-fold opening after about seven hours, with 50% of actuation occurring in the first 20 minutes. During the course of these measurements, the cell contraction in the longitudinal direction (l) was found to be negligible.

**Fig 7:**
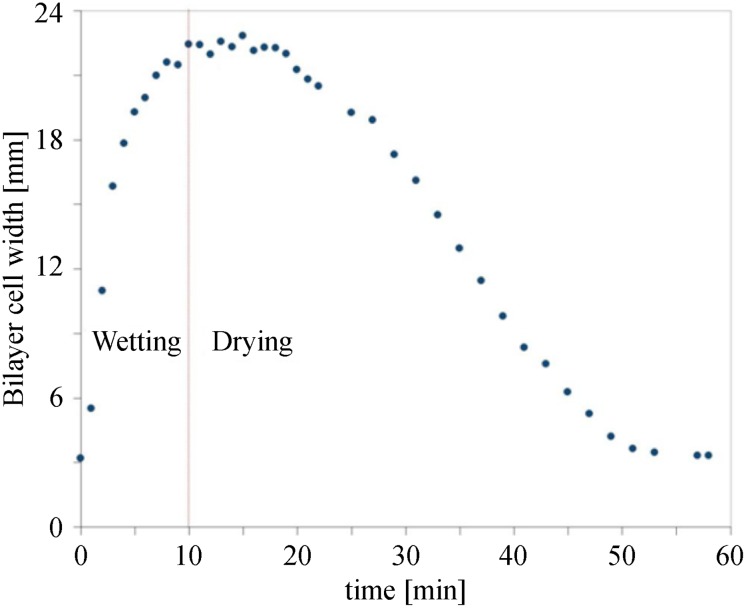
Reversible actuation of the bilayer-cell upon wetting-drying cycles. Opening and closing of a bilayer-cell upon wetting and drying at room temperature (50%RH) is plotted as a function of time.

A much more rapid actuation was observed upon spraying liquid water on the cells. The opening/closing of the cells was monitored upon wetting and the subsequent drying at 25°C and 50%RH (Fig 7). The cell’s opening started at ca. 0.5 minute after exposure to liquid water, and continued to an almost 8-fold opening of the cells in ca. 10 minutes. The bilayer-cells started to close within only a few minutes after the wetting was stopped and drying of the bilayers continued until the initial closed state of the cells was reached.

The equilibrium curvature of the actuated single bilayer and cell-wall bilayers was measured for both the wetting and relative humidity experiments (Table 3).

**Table 3:** Equilibrium curvature of the actuated bilayer and bilayer-cell upon exposure to 95% relative humidity and wetting with liquid water.

	κ_exp_ [m^-1^]	
	Bilayer	Bilayer-Cell
95% RH	33 ±1	25 ±1
WET	41 ±1	29 ±1

The equilibrium curvature measured in 95%RH was found to be less than the curvature of the same system actuated by wetting. Hydro-actuation is a time-dependent diffusion process, thus the temporal difference between the adsorption processes in the two experiments can explain the observed discrepancy, as the actuation in the humidity chamber might need longer time to achieve the equilibrium state.

**Fig 8:**
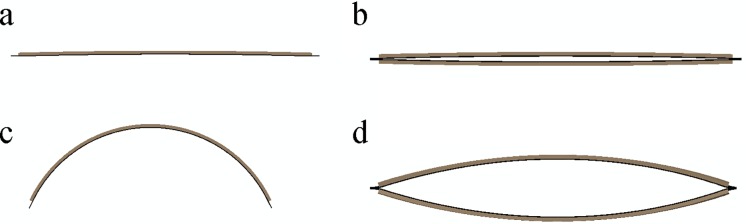
Finite element simulation of bilayer and bilayer cell. Left: single bilayer in initial (a) and actuated state (c); Right: Bilayer-cell in initial (b) and actuated (d) configuration.

Using the measured experimental parameters, Timoshenko's formula predicts a theoretical curvature of about κ_th_≅39 m^-1^, in agreement with the experimental values for single bilayers. The equilibrium curvature of the actuated bilayer-cell wall, on the other hand, was found to be less than that of the single bilayer, which can be attributed to the geometrical constraints due to the rotational rigidity of the bilayer-cell hinges. To verify this, finite-element analysis of the actuation of a similar bilayer-cell structure was performed considering the actual cell hinges geometry of the prototype. The FE simulation reproduced the opening of the cell upon actuation, resulting in a cell-wall curvature of about *κ*_*FE cell-wall*_ *≅ 13 m*^*-1*^ (Fig 8). The predicted curvature for the bilayer-cell model was significantly smaller than the single bilayer, which highlights the significant effect of the rotational rigidity at the hinges. While in the prototypes, the two bilayers are secured at the hinges, in the FE simulations the hinges are drawn as bulk bodies with rigid anchor points resulting in a higher rotational rigidity, which explains the smaller calculated curvature compared to the prototype cell-walls.

**Fig 9:**
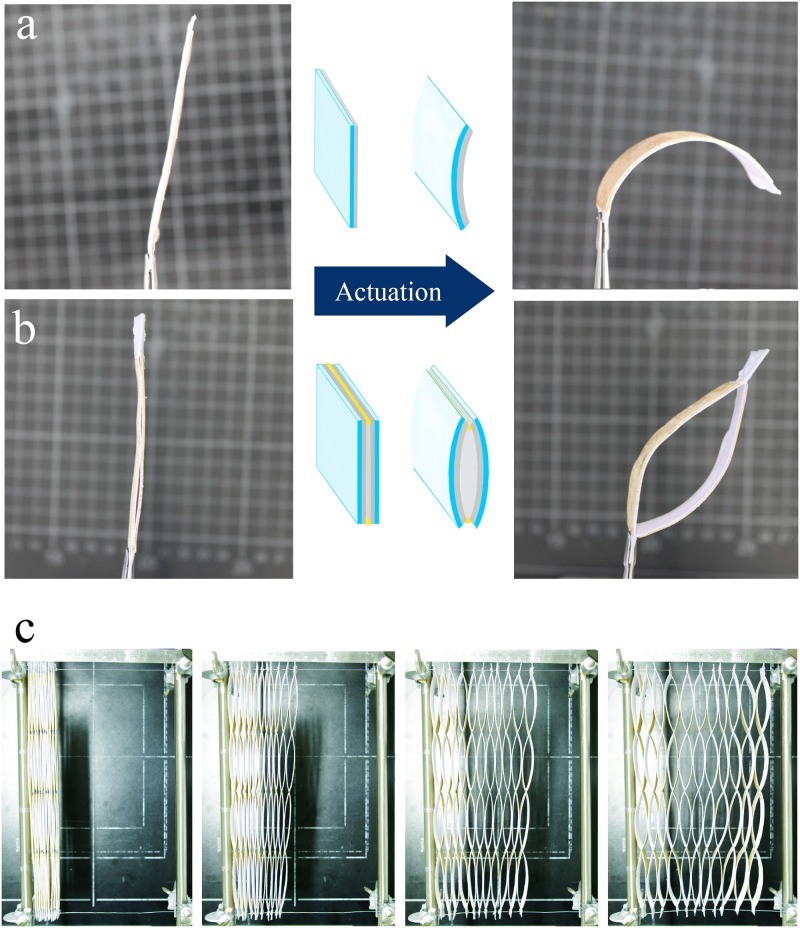
Passive hydro-actuation of bio-inspired bilayer-honeycomb device. Initial (left) and final actuated state (right) of the passive hydro-actuation upon changing the relative humidity from 50 to 95% are depicted at different levels of the design; (a) A bilayer made up of spruce veneer (active layer) glued to a thick paper (passive layer), bends upon anisotropic swelling of the spruce veneer in the direction perpendicular to the cellulose fibrils orientation. (b) Two of such bilayers attached together, constructs a cell-like structure that can open/closes upon changes in the relative humidity. (c) Scaling up the bilayer-cell concept into a hydro-actuated honeycomb prototype that expands up to 5 fold upon actuation (sequential images after 0, 2, 4 and 16 hours of exposure to 95%RH) [20].

By scaling up the bilayer-cells into a honeycomb structure, an autonomous hydro-responsive honeycomb device was constructed as a proof of concept (Fig 9, also see S2, S3 and S4 Movies).

The proposed design shows how the relatively small strain generated as the initial response of the material (anisotropic swelling of spruce veneers in the radial direction) can be translated through a simple yet efficient multi-scale architecture into a tailorable large deformation.

## Conclusions and Outlook

As we have shown, many design strategies can be extrapolated from a natural integrated hydro-responsive actuating device such as the ice plant seed capsule. These concepts can be directly implemented into an engineered equivalent at larger scales (as in the swelling honeycomb approach); or can be transferred to smaller scales of a larger device (as in the bilayer-honeycomb approach). One aspect that has to be considered when scaling up the size of such devices is that larger diffusion-based devices might necessitate longer actuation times. Thanks to their cellular structure, smart upscaling without loss of actuation speed could be achieved by increasing the number of actuation units (i.e. the number of cells in both honeycombs devices) rather than the characteristic length of the diffusive path (the inclusion size and bilayer thickness, respectively). Another advantage deriving from the cellular structure of such actuating devices is their modular nature. For example, one can envisage an integrated large scale device with multiple actuating portions. Thanks to the cellular structure, different portions can be integrated together through structural gradients (for example by changing the cell wall thickness), thus reducing interfacial stress concentration between the different regions and hence diminishing the risk of failure of the whole device.

Clearly, these considerations are application-specific and have to be made well in advance in the design process. More specifically, each of the proposed designs can be modified/optimized in a variety of ways.

The actuation capability of swelling-honeycomb actuators can be tailored by accessing multiple parameters, such as the mechanical properties of the materials, their geometrical arrangement and the sorbent-sorbate interaction [31]. To maximize the actuation potential of such a device, one could use forward osmotic swelling chambers [32] in the honeycomb cells in place of the polymeric swelling inclusions described here. Another remarkable feature of the swelling-honeycomb system is its versatility: for instance by simply using different inclusions/fillers in different cells or orchestrated insertion of defects (lattice dislocations) one could deliver a more complex spectrum of expansion behaviors embedded in the same honeycomb. Also, a specific recruitment pattern of the cells (as seen in [33]) could enable a reprogrammable actuation behavior. Finally, we showed how the actuation behavior of these swelling honeycombs remarkably depends on their geometric features and can be predicted by the proposed simplified model explicitly accounting for these features, enabling more freedom in design and prompt improvements of the same.

In the case of a bilayer-cell, a thorough material selection survey (different wood species, or other hydro- or thermo-responsive materials, etc.) can help to modify the design with a more suitable choice of active/passive layers depending on the desired function and actuating medium. In case of the wood/paper bilayer-honeycomb prototype, the supports at the connecting edges of the cells, and attachments between the cell rows can be improved (depending on the material and stimuli) to give a more robust structure. The total response can be enhanced by increasing the bending curvature through optimizing length, thickness and mechanical properties of the active and passive layer. In general and for any given bilayer actuator, the interfacial stress can be lowered by adding intermediate layers or a gradient between the most passive and the most active layer, thus lowering the risk of delamination.

Restricting the deformation of the honeycomb structure with a passive layer, either by attaching it to the edge of the cellular structure (like in the ice plant keels), or covering the face of the cells at one side, would result in a bilayer-honeycomb structure that flexes as a whole upon actuation. Such flexing structures (bilayer-honeycomb + covering substrate) can in principle be utilized as the walls of a larger actuating cell at a higher hierarchical level. Hence, conceptually one can think of further amplification of the response through design of higher levels of hierarchy on top of each other. Taking the same principles further, one can design structures with more sophisticated movements such as twisting [34].

Both proposed systems respond to an environmental change to perform mechanical work. One can speculate that the bilayer honeycomb system might be more suitable for applications in which large deformations are required, whereas the structure of the swelling honeycomb provides the basic load-bearing stability needed for force generation. Nevertheless, each system provides enough flexibility to explore a wide spectrum of responses: for example, in the bilayer-cell system, large deformations with low forces (or the opposite) can be generated by choosing thin (or thick) cell walls respectively. Although this comparison is not reported here, our paper provides the theoretical and experimental tools to investigate this further.

In general, different aspects of the actuated movement (amplitude, mode; bending, twisting etc.) can be tailored by varying different features of the system (geometrical features at various length scales, response to stimuli etc.) to develop “smart” actuation devices with a broad range of possible applications from facades and roofing systems to biomedical engineering.

In conclusion we emphasize on the versatility of both approaches, a common trait that eventually derives from the strong role of geometry.

## Supporting Information

S1 Movie Uniaxial expansion of honeycombs due to swelling of the inclusions. Local isotropic expansion of the inclusions is directed into uniaxial expansion of the whole honeycomb thanks to the honeycomb unit cell shape. The effect is more pronounced in a honeycomb with the same unit cell shape but thinner walls.
(AVI)

S2 Movie Hydro-responsive bilayer. The innert paper layer glued to a spruce veneer, restricts veneer’s anisotropic swelling in the direction perpendicular to the spruce cellulose fibrils orientation, which results in bending of the bilayer structure as a whole upon wetting-drying cycles.
(AVI)

S3 Movie Hydro-responsive bilayer-cell. Two spruce-paper bilayers attached together in a mirrored fashion, with the passive paper layers facing each other, constructs a cell-like structure that open-closes up to 8 folds upon wetting-drying cycles.
(AVI)

S4 Movie Hydro-responsive bilayer-honeycomb prototype. The hydro-actuated honeycomb’s walls are constructed from spruce veneer (active) and paper (passive) layers, with the passive paper layers facing the inside of the cells. Upon change of the environemnt realtive humidity from 50 to 95%, the bilayer walls bend and the whole honeycomb structure expands-contracts up to 5-folds.
(AVI)

## References

[1] Burgert I, Fratzl P. Actuation systems in plants as prototypes for bioinspired devices. Philos T R Soc A. 2009;367(1893):1541–57. 10.1098/rsta.2009.0003. WOS:000264660700005. 19324722

[2] Fratzl P, Barth FG. Biomaterial systems for mechanosensing and actuation. Nature. 2009;462(7272):442–8. 10.1038/nature08603. WOS:000272144200032. 19940914

[3] Razghandi K, Turcaud S, Burgert I. Hydro-Actuated Plant Devices. Nonlinear Elasticity and Hysteresis: Fluid-Solid Coupling in Porous Media. 2015:171–200. WOS:000359932800009.

[4] Dawson J, Vincent JFV, Rocca AM. How pine cones open. Nature. 1997;390(6661):668-. WOS:A1997YM50800024.

[5] Elbaum R, Gorb S, Fratzl P. Structures in the cell wall that enable hygroscopic movement of wheat awns. J Struct Biol. 2008;164(1):101–7. 10.1016/j.jsb.2008.06.008. WOS:000259660400013. 18625323

[6] Garside S, Lockyer S. Seed dispersal from the hygroscopic fruits of Mesembryanthemum carpanthea (Mesembryanthemum), pomeridiana N. E. Br. Ann Bot-London. 1930;44(175):639–U36. WOS:000200005700009.

[7] Lockyer S. Seed dispersal from hygroscopic Mesembryanthemum fruits, Bergeranthus scapigerus, schw, and dorotheanthus bellidiformis, n e br, with a note on Carpanthea pomeridiana, N E Br. Ann Bot-London. 1932;46(182):323–U15. WOS:000200006100006.

[8] Harrington MJ, Razghandi K, Ditsch F, Guiducci L, Rueggeberg M, Dunlop JWC, et al Origami-like unfolding of hydro-actuated ice plant seed capsules. Nat Commun. 2011;2 ARTN 337 10.1038/ncomms1336. WOS:000294804400003. 21654637

[9] Razghandi K, Bertinetti L, Guiducci L, Dunlop JWC, Fratzl P, Neinhuis C, et al Hydro-actuation of ice plant seed capsules powered by water uptake. Bioinspir Biomim Nan. 2014;3(3):169–82. 10.1680/bbn.14.00016. WOS:000341385900007.

[10] Timoshenko S. Analysis of bi-metal thermostats. J Opt Soc Am Rev Sci. 1925;11(3):233–55. 10.1364/Josa.11.000233. WOS:000200893300005.

[11] Burgert I, Fratzl P. Plants control the properties and actuation of their organs through the orientation of cellulose fibrils in their cell walls. Integr Comp Biol. 2009;49(1):69–79. 10.1093/icb/icp026. WOS:000267602100009. 21669847

[12] Erb RM, Sander JS, Grisch R, Studart AR. Self-shaping composites with programmable bioinspired microstructures. Nat Commun. 2013;4 ARTN 1712 10.1038/ncomms2666. WOS:000318872100069. 23591879

[13] Fratzl P, Elbaum R, Burgert I. Cellulose fibrils direct plant organ movements. Faraday Discuss. 2008;139:275–82. 10.1039/b716663j. WOS:000258949700017. 19049001

[14] Ma MM, Guo L, Anderson DG, Langer R. Bio-Inspired Polymer Composite Actuator and Generator Driven by Water Gradients. Science. 2013;339(6116):186–9. 10.1126/science.1230262. WOS:000313328200039. 23307738 PMC3635810

[15] Reyssat E, Mahadevan L. Hygromorphs: from pine cones to biomimetic bilayers. J R Soc Interface. 2009;6(39):951–7. 10.1098/rsif.2009.0184. WOS:000269197900011. 19570796 PMC2838359

[16] Shahinpoor M, Thompson MS. The Venus-Flytrap as a Model for a Biomimetic Material with Built-in Sensors and Actuators. Mat Sci Eng C-Biomim. 1995;2(4):229–33. 10.1016/0928-4931(95)00105-0. WOS:A1995TG13100009.

[17] Ruggeberg M, Burgert I. Bio-Inspired Wooden Actuators for Large Scale Applications. Plos One. 2015;10(4). ARTN e0120718 10.1371/journal.pone.0120718. WOS:000352139000031. 25835386 PMC4383548

[18] Guiducci L, Fratzl P, Brechet YJM, Dunlop JWC. Pressurized honeycombs as soft-actuators: a theoretical study (vol 11, 20140458, 2014). J R Soc Interface. 2014;11(101). ARTN 20141031 10.1098/rsif.2014.1031. WOS:000343672800027. PMC4233694 24966238

[19] Guiducci L, Weaver JC, Brechet YJM, Fratzl P, Dunlop JWC. The Geometric Design and Fabrication of Actuating Cellular Structures. Adv Mater Interfaces. 2015;2(11). ARTN 1500011 10.1002/admi.201500011. WOS:000358600100012.

[20] Razghandi K. Passive hydro-actuated unfolding of ice plant seed capsules as a concept generator for autonomously deforming devices [Berlin, Techn Univ, Diss, 2015]2015.

[21] Guiducci L. Passive biomimetic actuators: the role of material architecture. [PhD Thesis]. Potsdam: Universität Potsdam; 2014.

[22] Brown R. Physical Testing of Rubber: Springer US; 2006.

[23] Schneider CA, Rasband WS, Eliceiri KW. NIH Image to ImageJ: 25 years of image analysis. Nat Methods. 2012;9(7):671–5. 10.1038/nmeth.2089. WOS:000305942200020. 22930834 PMC5554542

[24] Caulfield DF, Gunderson D, editors. Paper testing and strength characteristics. 1988 Paper Preservation Symposium: Capital Hilton, Washington, DC, October 19–21; 1988: TAPPI Press.

[25] Neuhaus FH. Elastizitätszahlen von Fichtenholz in Abhängigkeit von der Holzfeuchtigkeit: Ruhr-Universität Bochum, Institut für Konstruktiven Ingenieurbau; 1981.

[26] Teraoka I. Polymer solutions in confining geometries. Prog Polym Sci. 1996;21(1):89–149. 10.1016/0079-6700(95)00018-6. WOS:A1996UC23200004.

[27] Treloar LRG. The physics of rubber elasticity. Oxford,: Clarendon Press; 1949 254 p. p.

[28] Kang MK, Huang R. A Variational Approach and Finite Element Implementation for Swelling of Polymeric Hydrogels Under Geometric Constraints. J Appl Mech-T Asme. 2010;77(6). WOS:000284078500005.

[29] Bertinetti L, Fischer FD, Fratzl P. Physicochemical Basis for Water-Actuated Movement and Stress Generation in Nonliving Plant Tissues. Phys Rev Lett. 2013;111(23). WOS:000328617100008. 10.1103/PhysRevLett.111.238001 24476305

[30] Rafsanjani A, Brule V, Western TL, Pasini D. Hydro-Responsive Curling of the Resurrection Plant Selaginella lepidophylla. Sci Rep-Uk. 2015;5 ARTN 8064 10.1038/srep08064. WOS:000348351800014. 25623361 PMC4306918

[31] Ionov L. Biomimetic Hydrogel-Based Actuating Systems. Adv Funct Mater. 2013;23(36):4555–70. 10.1002/adfm.201203692. WOS:000327493000014.

[32] Sinibaldi E, Argiolas A, Puleo GL, Mazzolai B. Another Lesson from Plants: The Forward Osmosis-Based Actuator. Plos One. 2014;9(7). ARTN e102461 10.1371/journal.pone.0102461. WOS:000339618600083. 25020043 PMC4097062

[33] Shepherd RF, Ilievski F, Choi W, Morin SA, Stokes AA, Mazzeo AD, et al Multigait soft robot. P Natl Acad Sci USA. 2011;108(51):20400–3. 10.1073/pnas.1116564108. WOS:000298289400035. 22123978 PMC3251082

[34] Turcaud S, Guiducci L, Fratzl P, Brechet YJM, Dunlop JWC. An excursion into the design space of biomimetic architectured biphasic actuators. Int J Mater Res. 2011;102(6):607–12. WOS:000292525800003.

